# A Rare and Late Complication of Subureteric Teflon Injection With Non-Animal Stabilized Hyaluronic Acid/Dextranomer Gel

**DOI:** 10.7759/cureus.33238

**Published:** 2023-01-01

**Authors:** Fatima Adamu-Biu, Eun Y Han, Sayed I Miakhil, Rohit Makhija

**Affiliations:** 1 Urology, North West Anglia NHS (National Health Service) Foundation Trust, Peterborough, GBR; 2 Surgery, North West Anglia NHS (National Health Service) Foundation Trust, Peterborough, GBR

**Keywords:** ureteric obstruction, ureteric reimplantation, subureteric injection, deflux, vesicoureteral reflux

## Abstract

Primary non-syndromic vesicoureteral reflux (VUR) is the commonest paediatric anomaly of the urinary tract. Complications of high-grade VUR include recurrent urinary tract infections, pyelonephritis, reflux nephropathy, and irreversible renal failure. The primary aim of its management centres on minimizing the number of urinary tract infections and renal scarring via surgical correction or continuous antibiotic prophylaxis. A rare complication of surgical treatment by subureteric Teflon injection with non-animal stabilized hyaluronic acid/dextranomer gel (NASHA/Dx) is ureteric obstruction. We report the case of a 38-year-old female who was diagnosed with ureteric obstruction secondary to subureteric injection with Deflux injection 30 years after endoscopic correction of VUR. She was successfully treated with ureteric reimplantation. Although considered efficient and safe, subureteric injection of bulking agent Deflux can be associated with delayed ureteric obstruction. This case highlights the need for long-term follow-up to allow timely detection and management of delayed ureteric obstruction. The possibility of late complication must also be addressed when obtaining pre-operative informed consent

## Introduction

Vesicoureteral reflux (VUR), a common paediatric anomaly with an estimated prevalence of 10%, can cause infection, scarring and end-stage renal disease [1]. According to the American Urological Association, relative indications for surgical intervention include high-grade reflux, older age at initial presentation, low probability of spontaneous resolution, breakthrough febrile urinary tract infections despite antibiotic prophylaxis, renal scarring, and parental preference [2]. The main goal of surgical interventions is to elongate the length of the intravesical ureter or alter the angle of the ureteral orifice and provide coaptation of the distal ureter during bladder filling [3]. Current surgical treatments of VUR include open, laparoscopic or robot-assisted ureteroneocystostomy, hydrodistension implantation, and endoscopic injection of the bulking agent.

Since the advent of subureteric Teflon injection (STING) by O’Donnell and Puri in 1984, it has been widely implemented [2]. In comparison to open ureteral reimplantation, STING provides significant advantages including a comparable success rate, shorter hospital stays, fewer complications, minimal cost, and reduced morbidity [4]. At present, non-animal stabilized hyaluronic acid/dextranomer gel (NASHA/Dx) represents the mainstay bulking agent and is regarded as the safest and most biocompatible material with the least potential for migration from the injection site [5]. Despite its safety profile and success, we report an unusual complication of STING with NASHA/Dx in a middle-aged female patient 30 years post intervention.

## Case presentation

**Figure 1:**
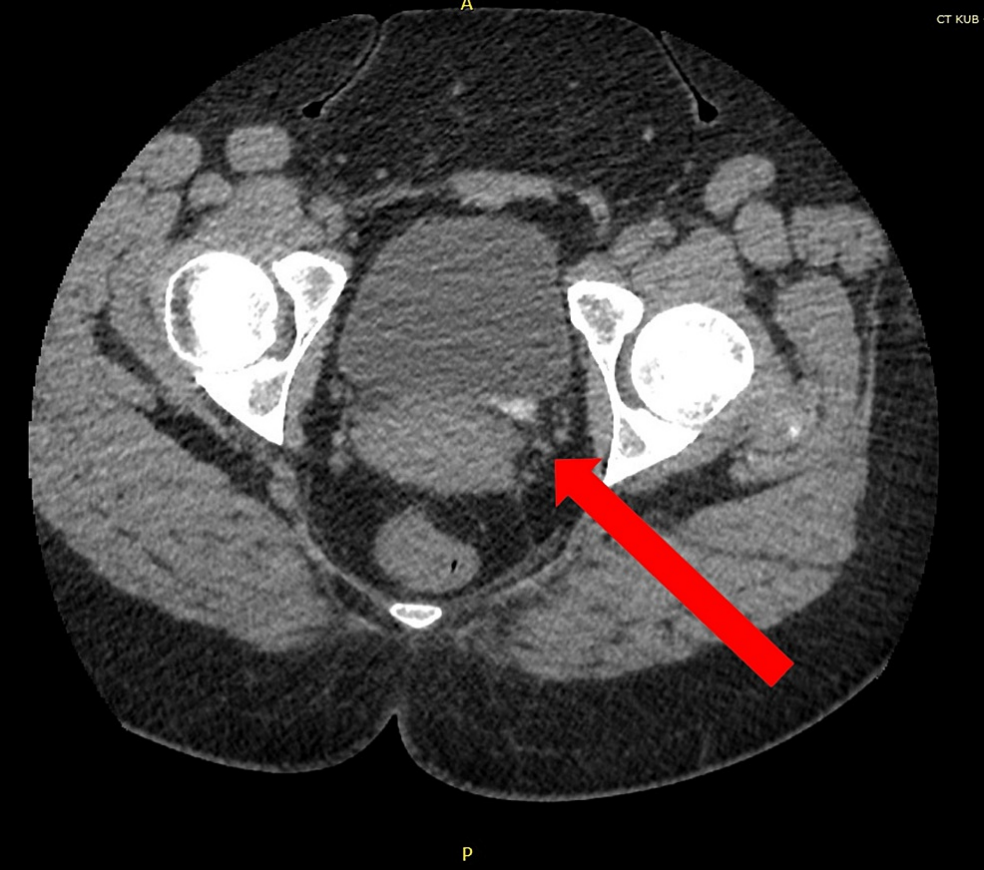
Unenhanced computed tomography scan of the kidneys, ureters, and bladder demonstrating hyperdense calcification at left vesicoureteric junction

**Figure 2:**
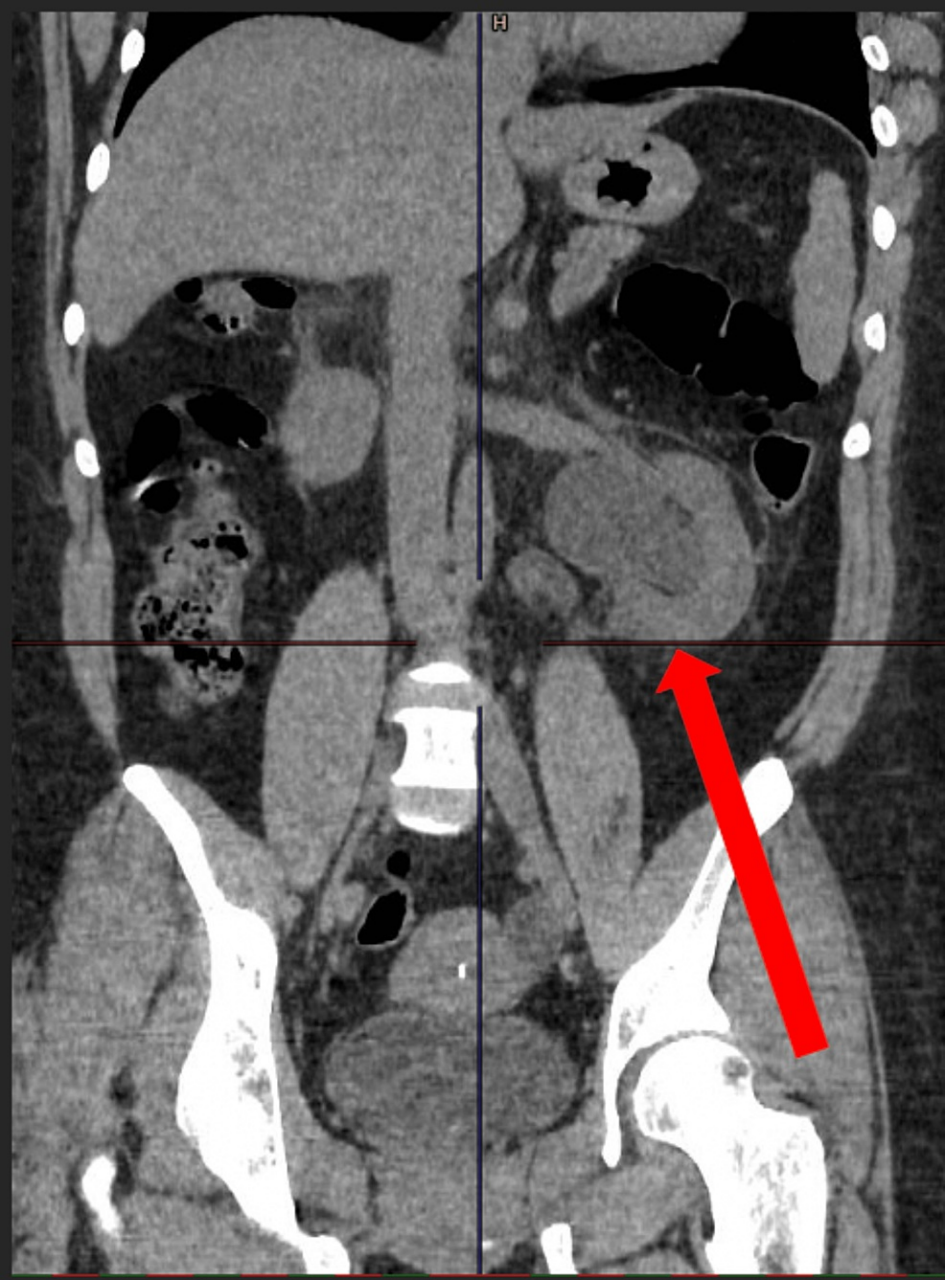
Unenhanced computed tomography scan of the kidneys, ureters, and bladder demonstrating left ureterohydronephrosis

A 38-year-old patient presented with a one-week history of left loin to groin pain. She had four prior presentations of urinary tract infections that were minimally responsive to antibiotics. At the age of one, she was diagnosed with congenital VUR and underwent a STING procedure with NASHA/Dx. During the initial investigation, urinalysis was positive for blood and urine culture was negative. Blood tests showed a white blood cell count of 20.0 x 10^9^/L, c-reactive protein of 35 mg/L, creatinine of 76 umol/L, urea of 3.2 mmol/L, and estimated glomerular filtration rate (eGFR) 86 ml/min. Unenhanced computed tomography scan of the kidneys, ureters, and bladder (CT KUB) demonstrated a high-density (19 mm x 8mm) calcification at the left vesicoureteric junction (Figure 1) with significant left ureterohydronephrosis (Figure 2).

At this stage, the main differential diagnosis included obstructing left vesicoureteric junction (VUJ) calculi or calcification of NASHA/Dx at VUJ from a previous STING procedure. 

**Figure 3:**
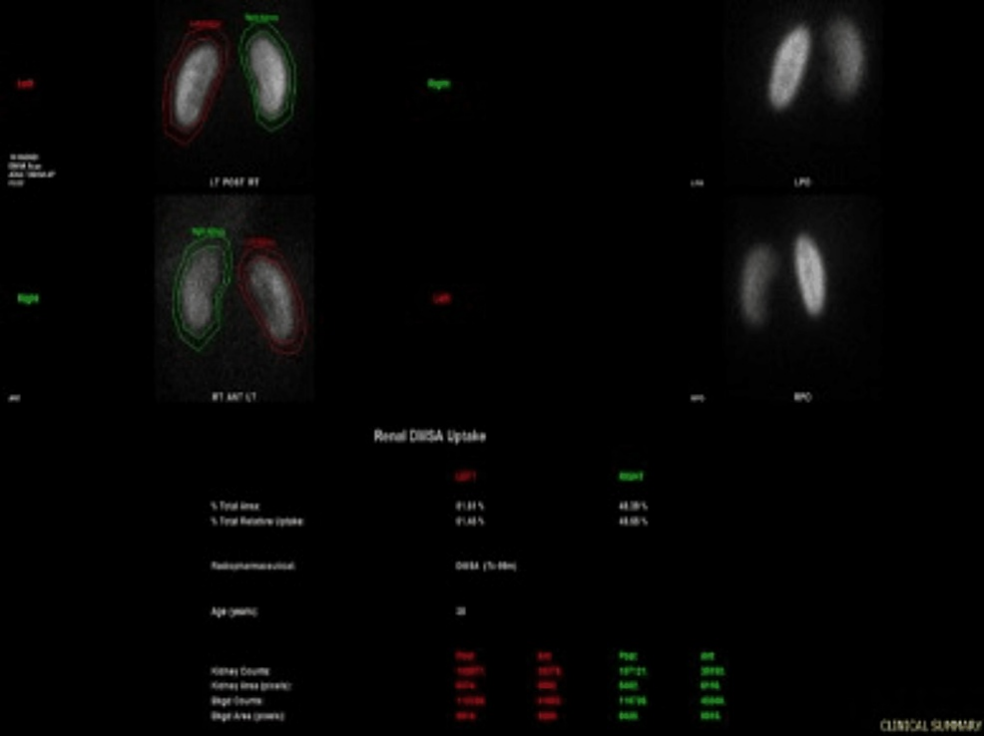
DMSA scan showing homogenous isotope uptake without focal scarring seen in both kidneys DMSA: Technetium-99m dimercaptosuccinic acid

To assess for parenchymal damage and differential kidney function [6], Technetiumc-99m dimercaptosuccinic acid (DMSA) scintigraphy was performed. Differential cortical tracer uptake of the left kidney and the right kidney was 51.45% and 48.55%, respectively (Figure 3). Despite the left VUJ obstruction and history of VUR, DMSA was normal with no obvious difference in uptake and cortical scarring between the left and right kidney. 

**Figure 4:**
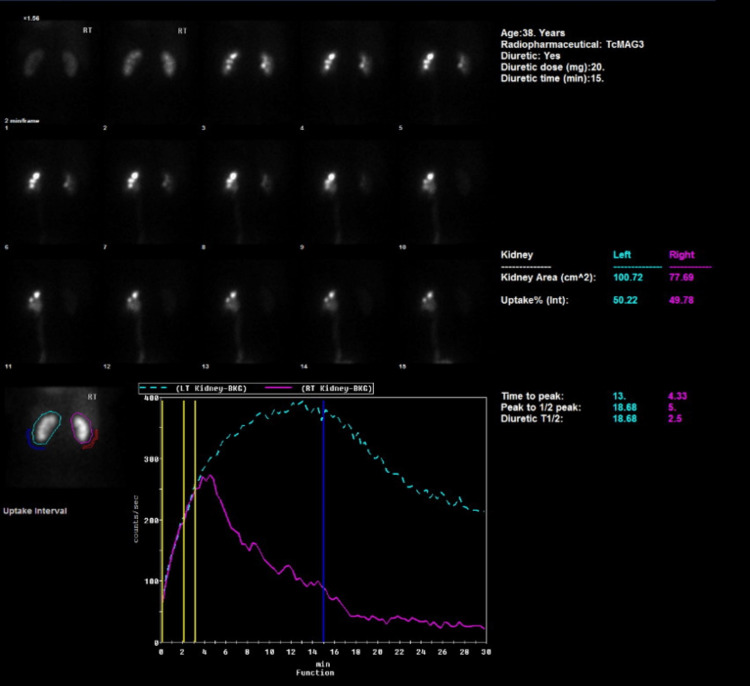
Pre-operative MAG-3 renogram. Left kidney (dotted blue line) illustrating an obstructive pattern of drainage with improvement in excretion with a diuretic (solid blue line), in comparison to the right kidney (solid pink line) MAG-3: Technetium-99m mercaptoacetyltriglycine

Technetium-99m mercaptoacetyltriglycine (MAG-3) renography was conducted to accurately assess the functional status and urodynamics of the left kidney. The left renogram curve demonstrated minimal drainage with mild response to diuretic administration, indicative of moderate excretion capacity despite the obstruction. The dynamic images reflected dilatation of the left pelvi-calyceal system and the entire length of the left ureter, which persisted 70 minutes post micturition (Figure 4). 

**Figure 5:**
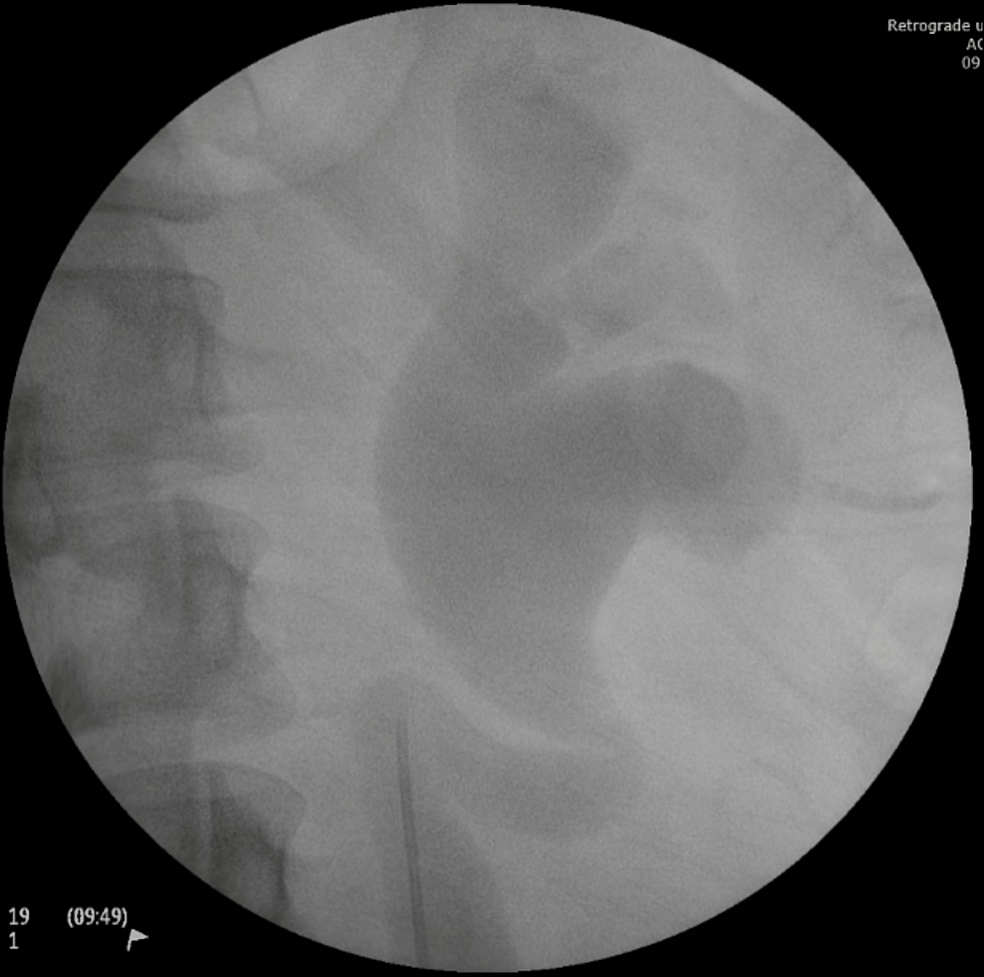
Retrograde pyelogram illustrating dilatation of left pelvi-calyceal system and ureter

To relieve the obstruction before definitive surgery, the patient underwent rigid cystoscopy and left ureteric stent insertion. Rigid cystoscopy showed the left ureteric orifice. A foreign material suggestive of NASHA/Dx at the left ureteric orifice was completely resected. Histopathological analysis of the foreign material revealed deposits of refractile substance provoking a giant cell response in keeping with reaction to NASHA/Dx injection. A retrograde pyelogram demonstrated a grossly dilated left pelvi-calyceal system and ureter (Figure 5). A ureteric stent was subsequently inserted under fluoroscopic guidance. 

**Figure 6:**
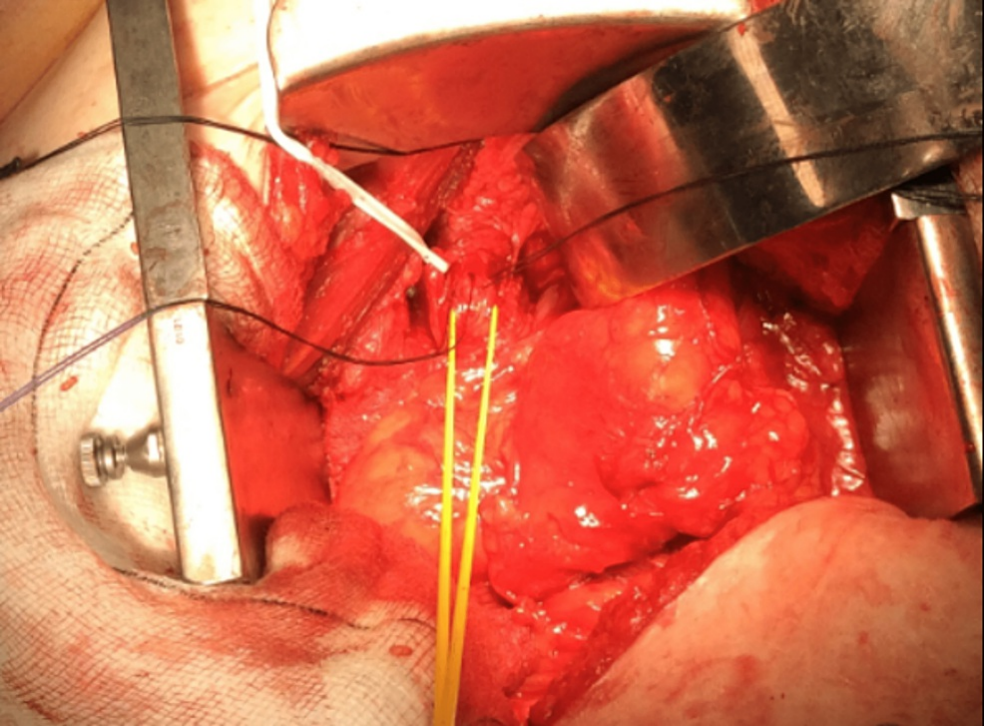
Identification of the left ureter by a yellow loop

To prevent further deterioration of the left kidney, the patient underwent left open ureteric reimplantation. A lower midline incision was made and the left ureter containing the previously inserted ureteric stent was identified (Figure 6). The left ureter was disconnected very close to the VUJ and was re-anastomosed into the bladder in a submucosal tunnel using 3-0 Vicryl sutures. A ureteric stent was temporarily inserted for 10 weeks to ensure adequate drainage in the postoperative period.

**Figure 7:**
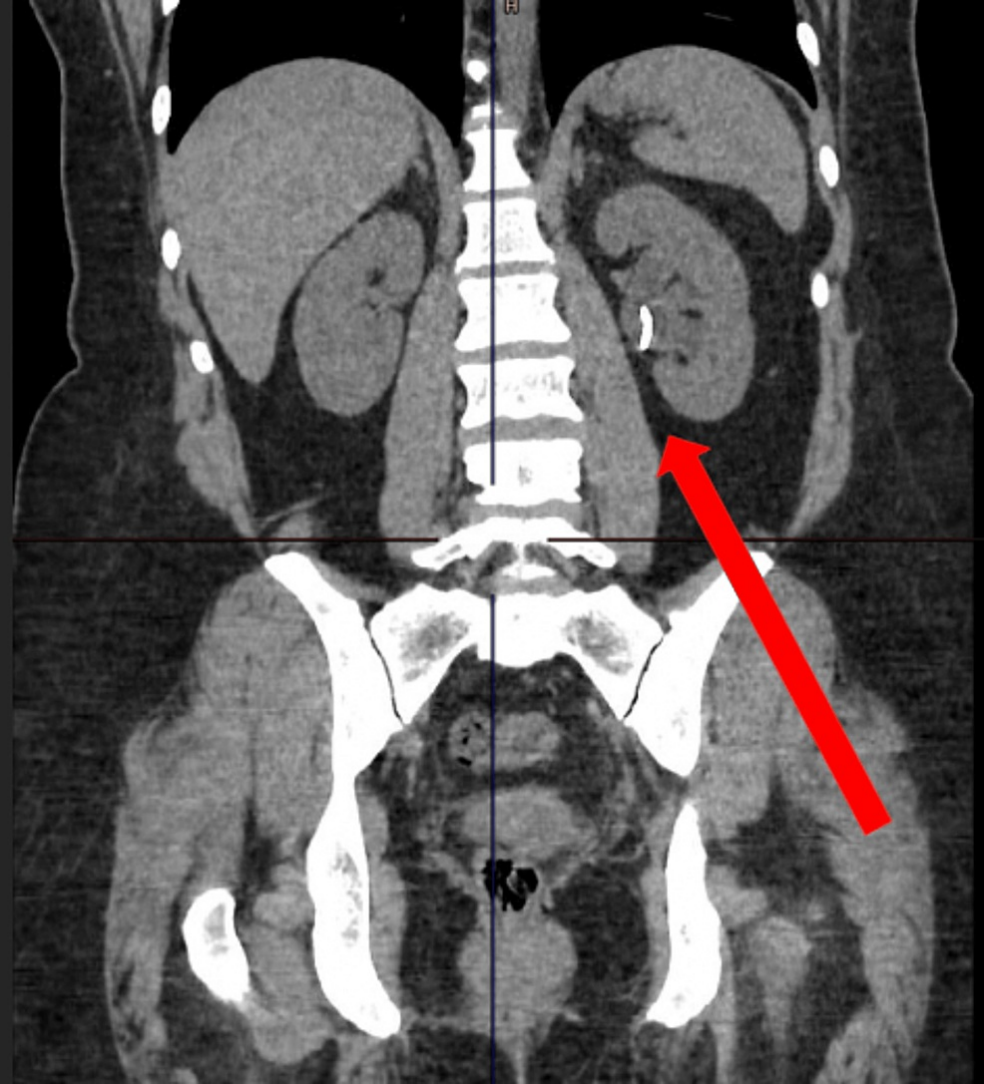
Computed tomography urogram showing resolution of the left hydroureteronephrosis

Three weeks postoperatively, a computed tomography urogram was performed. As shown in Figure 7, the previously observed left ureterohydronephrosis markedly improved. Normal time to bilateral corticomedullary opacification and contrast excretion was observed after contrast injection, representing successful ureteral re-implantation and intact renal function. 

**Figure 8:**
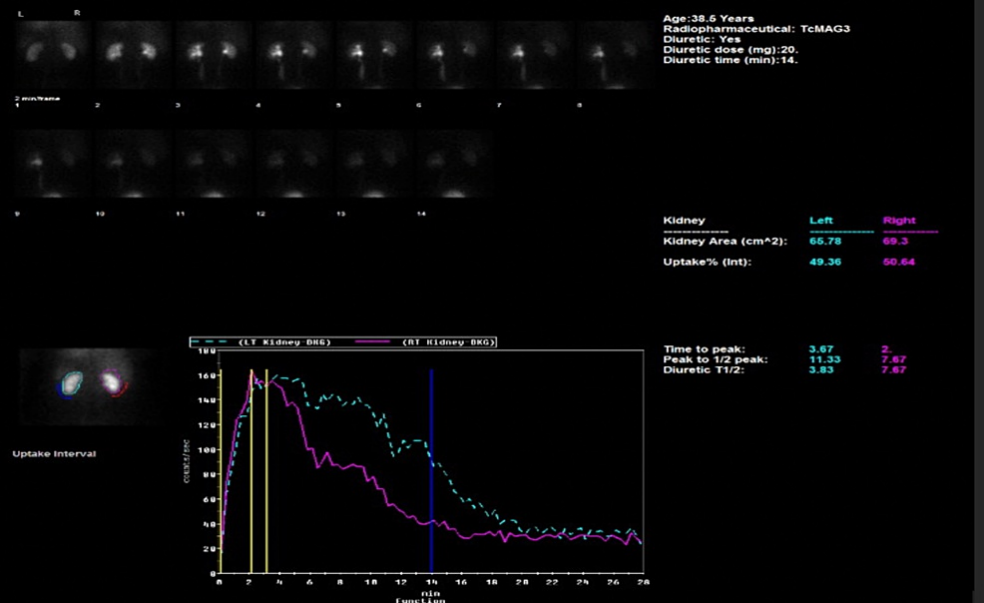
Postoperative MAG-3 renogram MAG-3: Technetium-99m mercaptoacetyltriglycine

Fifteen weeks postoperatively, an interval MAG-3 renogram was utilized to ascertain the excretive function of the left kidney. In comparison to the preoperative MAG-3 renogram in which the left kidney failed to drain until diuretic administration, the postoperative MAG-3 renogram revealed slow but immediate excretion followed by an exceptional response to a diuretic (Figure 8).

Dynamic images exhibited considerable improvement in the urodynamic status of the left kidney along with a complete resolution of ureteric obstruction and upstream hydroureteronephrosis.

## Discussion

VUR can be treated conservatively or surgically depending on its severity. Follow-up is not standardised but rather based on clinician preference and risk stratification [7]. A retrospective review showed that patients who had reimplantation of the ureter had a statistically significant greater chance of success compared with endoscopic injection [8]. Apart from complications like immune reaction and foreign body migration, ureteric obstruction is a rare but documented complication of STING procedure with a case of occurrence even up to 21 years later [9,10]. To our knowledge, our case represents the most delayed presentation of ureteric obstruction; 30 years post STING procedure. 

While ureteric obstruction secondary to STING has been reported to be managed endoscopically, there have also been reports of successful management of ureteric obstruction secondary to STING with ureteric re-implantation in a 25-year-old female who had STING at the age of 10 years [10]. Our case describes the successful management of ureteric obstruction secondary to STING with ureteric re-implantation in a patient more than 30 years post STING procedure. 

## Conclusions

This case of late ureteric obstruction up to 30 years later highlights the need for long-term follow-up in such patients, certainly much later than childhood. Furthermore, increased awareness of the delayed complication is warranted as its clinical presentation and imaging can closely mimic renal colic. Despite its effectiveness and simplicity, clinicians must provide extensive counselling preoperatively regarding the potential long-term implications of the STING procedure in adulthood.
